# Angiopoietin-Like Proteins 2 and 3 in Children and Adolescents with Obesity and Their Relationship with Hypertension and Metabolic Syndrome

**DOI:** 10.1155/2021/6748515

**Published:** 2021-08-12

**Authors:** Zahra Arab Sadeghabadi, Mitra Nourbakhsh, Mohammad Alaee, Mona Nourbakhsh, Seyedeh Sara Ghorbanhosseini, Roya Sharifi, Maryam Razzaghy-Azar

**Affiliations:** ^1^Metabolic Disorders Research Center, Endocrinology and Metabolism Molecular-Cellular Sciences Institute, Tehran University of Medical Sciences, Tehran, Iran; ^2^Department of Biochemistry, Faculty of Medicine, Iran University of Medical Sciences, Tehran, Iran; ^3^Finetech in Medicine Research Center, Iran University of Medical Sciences, Tehran, Iran; ^4^Hazrat Aliasghar Children's Hospital, Iran University of Medical Sciences, Tehran, Iran; ^5^Department of Biochemistry, Faculty of Pharmacy, Isfahan University of Medical Sciences, Isfahan, Iran; ^6^Department of Medical Laboratory Sciences, School of Allied Medical Sciences, Iran University of Medical Sciences, Tehran, Iran

## Abstract

**Background:**

Angiopoietin-like protein 2 (ANGPTL2) is one of the adipocyte-derived inflammatory factors which connects obesity to insulin resistance. ANGPTL3 has a direct role in regulation of lipid metabolism. The objective of this study was to evaluate ANGPTL2 and ANGPTL3 in childhood obesity and their relationship with metabolic syndrome.

**Methods:**

70 children and adolescents, 35 obese and 35 normal-weight subjects, were enrolled in this research after complete clinical examination and anthropometric evaluations. Serum ANGPTL2 and ANGPTL3 and insulin were measured by enzyme-linked immunosorbent assay (ELISA). Homeostatic model assessment of insulin resistance (HOMA-IR) was calculated and used to estimate insulin resistance (IR). Colorimetric methods were used for the assessment of fasting plasma glucose (FPG), LDL-C, HDL-C, total cholesterol (TC), and triglyceride (TG).

**Results:**

The levels of ANGPTL2 and ANGPTL3 were significantly higher in obese subjects than those in controls, but they did not differ significantly in subjects with or without IR. ANGPTL3 was found to be significantly elevated in obese children with metabolic syndrome (MetS) in comparison with those without MetS. Both of the studied ANGPTLs were positively correlated with BMI, systolic blood pressure (SBP), diastolic blood pressure (DBP), TC, and LDL-C. The correlation between ANGPTL3 and either TC or LDL-C remained significant after adjusting for BMI.

**Conclusion:**

Serum ANGPTL2 and ANGPTL3 were elevated in obesity and associated with blood pressure and indices of metabolic syndrome, suggesting that they might be involved in the advancement of obesity-related hypertension and metabolic syndrome.

## 1. Introduction

Obesity is currently considered a global burden with increasing prevalence worldwide [1]. Obesity is the main risk factor for long-term disorders, including metabolic syndrome (MetS), cardiovascular disease, and type 2 diabetes mellitus [2]. Childhood obesity is especially of growing concern because health condition in childhood dramatically affects adulthood life, and obese children are at higher risk for obesity-related disorders at younger ages [3]. Obesity in children, especially metabolically unhealthy phenotype and insulin resistance, negatively affects cardiovascular remodeling which results in subclinical myocardial dysfunction [4].

Adipose tissue has been recognized as one of the critical organs that regulate systemic metabolism [5]; thus, obesity is generally accompanied by remarkable alterations in different adipokines and hormones, which per se affect various enzymes and several metabolic pathways and derange their homeostasis [6–8]. The alteration of adipokines has been observed in childhood obesity and has been blamed for the early appearance of cardiometabolic complications of obesity [9].

Angiopoietin-like (ANGPTL) proteins are secreted substances that are structurally similar to the angiopoietin family of proteins. Although several studies show that ANGPTLs potently regulate angiogenesis [10], some also possess pleiotropic activities such as inhibition of lipoprotein lipase and endothelial lipase, augmentation of energy expenditure, and induction of inflammation [11].

ANGPTL2 was first reported as a secreted protein with a weak stimulatory effect on endothelial cell sprouting in vitro [12]. This protein is a circulating glycoprotein abundantly expressed in adipose tissues. Both the expression and serum concentration of ANGPTL2 are increased in obese mice [13]. ANGPTL2 plays pivotal roles in various inflammatory diseases such as vascular inflammation, obesity, insulin resistance, and atherosclerosis [13, 14].

Treatment of diabetic mice with recombinant ANGPTL2 amplified insulin resistance [15], while genetic deletion of this protein improved adiposity and systemic insulin resistance [13]. In vivo, elevated serum ANGPTL2 levels positively associate with the development of type 2 diabetes [16]. Systemic levels of this protein are increased in the obese patients compared to healthy controls and are positively correlated with body mass index and insulin resistance [17].

ANGPTL3, mainly produced in the liver, is one of the essential regulators of lipid metabolism by inhibiting lipoprotein lipase (LPL). Lipolysis of triglycerides by LPL is the first step in chylomicron/very-low-density lipoprotein (VLDL) clearance at the luminal surface of the capillaries [18]. Therefore, ANGPTL3 deficiency leads to reduced cholesterol and triglycerides, and its loss-of-function mutation in mice results in hypolipidemia [19]. In humans, the complete absence of ANGPTL3 results in an increased LPL activity and low circulating free fatty acid levels [20]. Human ANGPTL3 also inhibits phospholipase activity of endothelial lipase, decreasing HDL levels due to elevated hydrolysis of HDL [21]. Thus, ANGPTL3 is linked tightly to lipid metabolism, and its aberrant expression has been linked to obesity and type 2 diabetes [22].

With the purpose of creating better clinical insights into obesity, the causal molecular aberrations should be explored. In our previous study, we investigated the relationship between childhood obesity and serum ANGPTL4 and demonstrated that ANGPTL4 levels are decreased in obese children compared to normal ones [23]. In the current study, we aimed to examine the probable relationship of serum ANGPTL2 and ANGPTL3 levels with obesity and its relevant metabolic parameters in children.

## 2. Materials and Methods

### 2.1. Subjects

Seventy children and adolescents, between 8 and 16 years of age, were enrolled in this research, selected through simple sampling based on the inclusion and exclusion criteria. Sample size was estimated according to the results of previous studies [24, 25], at 90% power and alpha level set at 0.01, with MedCalc software version 18.9.10 (Ostend, Belgium). A complete history and clinical checkup was carried out for all the participants. Subjects in both groups did not show any clinical or laboratory signs of any disease and were not taking any medications or supplements. Some of the cases were taking multivitamins, and these cases were not excluded from the study. The subjects were not under any particular nutritional intervention or any diet and they followed their normal life pattern. Additionally, the subjects were not participating in any vigorous exercise or involved in professional sport. Therefore physical activity and diet of all the subjects were considered to be normal. Any subject that did not match the above criteria was excluded from the study.

The patients were examined and their history was taken. Weight, height, and waist and hip circumference were recorded on the day of sample collection. Body mass index (BMI) was calculated (kg/m^2^), and BMI z-score was determined using the relevant calculator of Medscape (https://reference.medscape.com). The BMI percentiles were defined by the growth charts of US Centers for Disease Control and Prevention (CDC) 2000, according to the sex and age of each subject. Allocation of the subjects to the case and control groups was performed based on the BMI percentiles. Children with BMI above the 95th percentile were considered as the subjects with obesity, and those with BMI between the 5th and 85th percentiles were categorized as the control group. The two groups were matched according to sex and age. Underweight subjects with BMI lower than the 5th percentile and overweight subjects with BMI percentile between the 85th and 95th percentiles were excluded.

Blood pressure (systolic: SBP and diastolic: DBP) was determined by a single clinician, using a Mercury sphygmomanometer based on the fourth report of NHLBI on the diagnosis, evaluation, and treatment of high blood pressure in children and adolescents [24]. Waist circumference (WC) was measured according to the WHO STEPS protocol at the approximate midpoint between the lower margin of the last palpable rib and the top of the iliac crest. Hip (HC) circumference was measured around the widest portion of the buttocks [25]. These measurements were performed by stretch‐resistant tape snug around the body but not pulled so tight, with the subjects being in the standing position. WC percentiles were evaluated based on the age and gender of the subjects [26].

The study and the sample collection procedure were explained for children and their parents or guardians who accompanied the children. Written informed consent was taken from participants and all of the parents/guardians. This study followed the ethical rules for medical research involving human subjects of the Declaration of Helsinki (1964), and the study received approval of the Ethics Committee of Endocrinology and Metabolism Research Institute, Tehran University of Medical Sciences.

### 2.2. Biochemical Measurements

Samples from the venous blood were taken after an overnight fast of 8–12 hours in the morning. Blood samples were centrifuged, and the separated plasma and serum samples were kept at −80°C for future analyses. The measurement of fasting plasma glucose (FPG), HDL-C, low-density lipoprotein cholesterol (LDL-C), total cholesterol (TC), and triglyceride (TG) was performed using enzymatic assay kits (Pars Azmoon, Tehran, Iran). The concentration of insulin in plasma was measured by enzyme-linked immunosorbent assay (ELISA) by a specific kit (Monobind Inc., CA, USA), with sensitivity of 0.182 *μ*IU/ml and correlation coefficient of 0.975 for accuracy. The intra- and interassay coefficients of variation (CV) for precision were less than 9 and 12, respectively.

### 2.3. Definition of Insulin Resistance and Metabolic Syndrome

Estimation of insulin resistance (IR) was performed using homeostatic model assessment of insulin resistance (HOMA-IR), which was calculated by the following formula: serum insulin (*μ*IU/ml) × FPG (mg/dl)/405 [27]. The cutoff point of 3.16 for HOMA-IR was considered for the discrimination of children and adolescents harboring insulin resistance [28].

Metabolic syndrome (MetS) was diagnosed according to the criteria of the International Diabetes Federation (IDF) consensus guidelines for children and adolescents [29], because it has been shown that the definition proposed by IDF is more straightforward, accessible, and practical and is best applied for clinical use [30]. Based on these criteria, the subjects with WC being above the 90th percentile for their age and sex, representing abdominal obesity, together with two or more of the other parameters of MetS such as high SBP or DBP, FPG, TG, or low HDL-C, were considered as having MetS.

### 2.4. Determination of Plasma ANGPTL2 and ANGPTL3 Concentrations

ELISA kits containing specific antibodies for ANGPTL2 (Cusabio, China) and ANGPTL3 (RayBiotech, USA), with the sensitivity of 0.39 ng/ml and 8.2 pg/ml, respectively, were used for measuring the levels of these two proteins in the plasma samples. The intra- and interassay CV values were <10 and < 12% for the ANGPTL2 kit and <8 and < 10% for the ANGPTL3 kit, respectively.

### 2.5. Statistical Analysis

The normal distribution of the resulting data was assessed using Kolmogorov-Smirnov test. Data is presented as mean ± standard deviation (SD) for parametric variables and median (interquartile range) for nonparametric variables. For the analysis of the differences between the obese and control groups, independent-samples *t-*test and Mann-Whitney *U* tests were used for parametric and nonparametric variables, respectively. Pearson's and Spearman's correlation tests were used to determine the relationship between variables. Partial correlation was applied to adjust for confounding factors. The statistical evaluations were carried out using SPSS software version 22.0 (SPSS, Inc., Chicago, IL, USA) and MedCalc software version 18.9.10 (Ostend, Belgium).

## 3. Results

The anthropometric features and biochemical parameters of the studied subjects are shown in Table 1. As the cases were matched, no significant difference was seen for gender distribution and the age of the subjects in the two studied groups. Weight, BMI, and its z-scores, WC, and SBP were significantly higher in obese children than in the normal-weight children and adolescents. Among the biochemical parameters, FPG, TG, LDL-C, insulin, and HOMA-IR showed elevation in obese group. Furthermore, HDL-C level was significantly lower in obese group than in the control group. Other studied parameters were not significantly different between obese and control groups.

According to HOMA-IR levels, 18.6% of the obese subjects were found to have insulin resistance, and 11.4% were identified as having MetS. Nonetheless, normal-weight subjects did not have any characteristics of MetS, and all of them had normal sensitivity to insulin.

**Figure 1:**
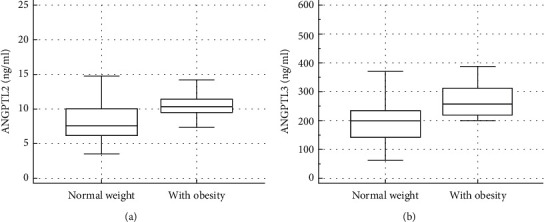
Comparison of (a) ANGPTL2 and (b) ANGPTL3 between subjects with obesity and those with normal weight.

Serum levels of ANGPTL2 and ANGPTL3 were significantly higher in obese children as compared with the normal-weight group (Figure 1; Table 1). When obese children were categorized as with or without IR or MetS, no statistically significant difference was seen for ANGPTL2. Nevertheless, ANGPTL3 level was significantly increased in obese subjects with MetS compared to children without MetS (306.06 ± 49.1 versus 226.13 ± 82.9 ng/ml, respectively; *P* = 0.01). The above parameters were not remarkably different between male and female subjects and did not correlate with age.

Correlation analysis revealed a notable positive relationship between plasma levels of ANGPTL2 and ANGPTL3 (*r* = 0.563, *P* = 0.01). The association of ANGPTL2 and ANGPTL3 with other variables is shown in Table 2. ANGPTL2 had significant correlations with TC and LDL, as well as with SBP and DBP. Additionally, a correlation between ANGPTL2 and BMI was also observed, which was significant. ANGPTL3 had correlations with BMI, BMI *z*-score, TC, LDL, SBP, and DBP. Furthermore, ANGPTL3 showed a significant relationship with WC and HC, as well as WC/HC ratio.

Correlations were also analyzed in separate groups of obese and normal-weight subjects, and both ANGPTL2 and ANGPTL3 were still significantly correlated with SBP and DBP. On the other hand, in normal-weight subjects, ANGPTL2 exhibited a remarkable negative correlation with insulin (*r* = -0.384, *P* = 0.023) and HOMA-IR (*r* = −0.356, *P* = 0.036). A similar correlation with insulin and HOMA-IR was also found for ANGPTL3 (*r* = −0.373, *P* = 0.027 and *r* = -0.360, *P* = 0.033, respectively) only in the control group.

Partial correlation was used to analyze the relationship between various parameters considering BMI as a confounding factor. The correlation between both studied ANGPTLs with TC and LDL-C remained significant after adjusting for BMI.

## 4. Discussion

In this study, we demonstrated that obese children had a significantly higher circulatory level of ANGPTL2 and ANGPTL3. ANGPTL 2 is mainly produced in adipose tissue, and its levels are directly related to the amount of fat and BMI index in mice and humans. Studies have shown that reduction of adiposity immediately resulted in a decline of ANGPTL2 [13]. In the early phase of obesity, adipose tissue expansion and adipocyte hypertrophy are physiological responses to the need to store excess lipids into adipocytes. ANGPTL2 may contribute to adipose tissue remodeling by promoting angiogenesis, macrophage recruitment, and extracellular matrix transformation [13]. ANGPTL2 has not been previously studied in obese children; however, elevated level of ANGPTL2 has been reported in obese women, which was consistent with our findings [17]. Maintaining adipocytes under conditions similar to the microenvironment of obese adipose tissue induced ANGPTL2 gene expression and secretion [17]. Enhanced expression of ANGPTL2 seems to be a response to the increased inflammation that accompanies obesity, such that TNF*α* and TGF*β* that are increased in obesity directly increase gene expression of ANGPTL2 [31].

ANGPTL3 was also found to be increased in obese children and adolescents. Its significant correlation with BMI and WC further confirmed the association between ANGPTL3 and obesity. In line with our findings, ANGPTL3 has been shown to be increased in obese subjects [32]. Some conflicting results also exist, which express no significant difference between overweight and normal-weight subjects, and it may be the reason of including overweight subjects (BMI > 85th percentile) and not just obese subjects [33]. ANGPTL3 has been introduced as a protein related to body fat mass and inflammation [34]. Additionally, ANGPTL3 is typically downregulated by leptin and insulin, and, therefore, resistance to leptin and insulin that generally happens in obesity may be responsible for its elevated levels [21]. The increased concentration of ANGPTL3 in obesity may also be due to the reduced level of some microRNAs such as miR-181d that inhibits the expression of ANGPTL3 [16].

ANGPTL2 and ANGPTL3 equally showed a significant correlation with SBP and DBP independent of BMI. ANGPTL3 seems to affect the arterial thickness and macrophage activity. It can bind to integrins, which have been proven to be strongly involved in atherosclerotic plaque formation [35]. ANGPTL3 deficiency has been linked to protection from coronary artery disease [36].

There are also several lines of evidence supporting the participation of ANGPTL2 in atherosclerosis and heart failure [37]. ANGPTL2 was first reported as a secreted protein with a weak stimulatory effect on endothelial cell sprouting in vitro [12]. This protein regulates angiogenesis similar to several other ANGPTLs. However, ANGPTL2 has the unique capacity to induce an inflammatory response in blood vessels [13]. It has been proven to be capable of accelerating vascular inflammation by triggering proinflammatory pathways in endothelial cells and enhancing macrophage infiltration, which results in endothelial dysfunction and atherosclerosis development [14]. ANGPTL2 has been connected to major adverse cardiovascular events in patients with diabetes [38] and has been introduced as a novel risk factor in developing CVD in the general population [39]. Thus, increased ANGPTL2 and ANGPTL3 levels in obese children and adolescents might be considered a risk factor that predisposes them to early cardiovascular disorders.

Another factor linking ANGPTLs to cardiovascular disorders is their role in lipid metabolism. In the current study, we found a positive correlation between ANGPTLs and TC as well as LDL-C. Consistently, previous in vitro and in vivo studies provided evidence for the causative influence of ANGPTLs on cholesterol and LDL metabolism.

Complete loss of ANGPTL3 leads to familial combined hypolipidemia characterized by low levels of LDL-C, and its silencing in mouse models causes a remarkable reduction in LDL-C, an effect linked to LDL receptor [40]. In dyslipidemic mice, inhibition of ANGPTL3 with evinacumab, an antibody against ANGPTL3, caused a dramatic decrease in atherosclerotic lesion area and necrotic content in animal models of dyslipidemia. Moreover, evinacumab could diminish LDL-cholesterol levels up to 23% in human subjects [41].

ANGPTL2 has also been connected to endothelial dysfunction and cardiovascular disorders through modulation of LDL metabolism. It has been reported that intravenous administration of ANGPTL2 in preatherosclerotic mice increased total cholesterol and LDL-cholesterol levels and intensely enhanced the expression of proinflammatory cytokines and accelerated atherosclerotic lesion formation [42].

ANGPTL2 causes insulin resistance (IR) in adipose tissue [43] and is associated with HOMA-IR in obese women [44]. Additionally, its knockdown has been proved to be beneficial for insulin responsiveness [45]. The relationship of ANGPTL3 with IR has also been previously reported [46], and its deficiency in both mice and humans has been linked to increased insulin sensitivity [47]. However, in the present study, we did not find any correlation between ANGPTLs 2 and 3 and indices of IR in obese subjects, and they were not significantly different between insulin-resistant and insulin-sensitive subjects. This discrepancy might be due to the low number of obese subjects with IR and the modest IR state.

Our findings showed a strong positive correlation between ANGPTL2 and ANGPTL3, independent of obesity and BMI status. The correlation between ANGPTL2 and ANGPTL3 proposes a possible reciprocal regulatory relationship between these two proteins, which needs to be further evaluated through in vitro studies.

The limitation of our study was that we could not perform an analysis of HOMA-IR and insulin resistance based on the puberty state of the subjects, due to the lack of data of pubertal staging. Thus, the relationship between ANGPTL2 and ANGPTL3 and the studied parameters considering puberty requires further studies.

The results of this study indicate a relationship between these proteins and blood pressure, suggesting their involvement with obesity-associated endothelial dysfunction. Evaluation of major parameters of cardiovascular dysfunction concerning ANGPTL2 and ANGPTL3, especially in a cohort design, is highly recommended for future research to establish the connection between these markers and risk of cardiovascular diseases that generally accompany obesity.

## 5. Conclusion

Our results provide evidence that obese children and adolescents encounter higher circulatory levels of ANGPTL2 and ANGPTL3 compared to normal-weight children, which might be associated with MetS and endothelial dysfunction. Further prospective studies are necessary to evaluate the link between ANGPTL2 and ANGPTL3 concentrations and cardiovascular outcomes of obesity.

## Figures and Tables

**Table 1:** Anthropometric and biochemical characteristics of the children and adolescents in the control and obese groups.

	Control	Obese	*P* value
Female/male	16/19	12/23	n.s.
Age (years)	11.11 ± 2.3	11.17 ± 2.3	n.s.
BMI (Kg/m2)	18.01 ± 1.9	29.67 ± 4.9	<0.001
BMI z-score	0.14 ± 0.7	2.2 ± 0.3	<0.001
WC (cm)	67.47 ± 8.4	93.82 ± 11.3	<0.001
HC (cm)	78.03 ± 7.5	100.13 ± 10.7	<0.001
WC/HC ratio	0.85 ± 0.05	0.93 ± 0.06	<0.001
SBP (mmHg)	112.51 ± 10.28	127.37 ± 15.38	<0.001
SBP *z*-score	-0.13 ± 0.6	1.5 ± 0.7	<0.05
DBP (mmHg)	73.4 ± 7.7	80.46 ± 9.5	<0.001
DPB *z*-score	-0.2 ± 0.3	0.93 ± 1.2	n.s.
FPG (mg/dl)	86.95 ± 6.5	93.7 ± 5.5	<0.001
TG (mg/dl)	81.08 ± 39.6	105.97 ± 41.9	<0.001
TC (mg/dl)	151.68 ± 27.3	161.74 ± 25.62	n.s.
LDL-C (mg/dl)	74.08 ± 13.27	81.94 ± 16.56	<0.05
HDL-C (mg/dl)	53.17 ± 12.7	47.17 ± 8.9	<0.05
Insulin (*µ*IU/dl)	6.5 ± 3.1	14.05 ± 9.3	<0.001
HOMA-IR	1.4 ± 0.7	3.2 ± 2.17	<0.001
ANGPTL2 (ng/ml)	7.6 (6.2–10.03)	10.32 (9.53–11.42)	<0.001
ANGPTL3 (ng/ml)	198.82 (142.2–233.2)	256.48 (219.7–311.8)	<0.0001

Data are presented as mean ± standard deviation (SD) for parametric variables and median (interquartile range) for nonparametric variables. BMI : body mass index; WC : waist circumference; HC : hip circumference; SBP: systolic blood pressure; DBP: diastolic blood pressure; FPG: fasting plasma glucose; TG: triglycerides; TC: total cholesterol; LDL-C : low-density lipoprotein cholesterol; HDL-C : high-density lipoprotein cholesterol; HOMA-IR : homeostasis model assessment-insulin resistance; ANGPTL: angiopoietin-like protein; n.s.: nonsignificant.

**Table 2:** Correlation coefficients of ANGPTL2 and ANGPTL3 plasma level with anthropometric and biochemical parameters.

Variable	ANGPTL2	ANGPTL3
BMI	0.235^*∗*^	0.392^*∗∗*^
BMI *z*-score	0.186	0.363^*∗∗*^
WC	0.165	0.347^*∗∗*^
HC	0.225	0.301^*∗*^
WC/HC ratio	0.023	0.303^*∗*^
SBP	0.237^*∗*^	0.406^*∗∗*^
DBP	0.247^*∗*^	0.432^*∗∗*^
FPG	0.127	0.194
TG	0.063	0.199
TC	0.271^*∗*^	0.241^*∗*^
LDL-C	0.279^*∗*^	0.239^*∗*^
HDL-C	0.098	0.002
Insulin	−0.003	0.096
HOMA-IR	0.008	0.104

BMI : body mass index; WC : waist circumference; HC : hip circumference; SBP: systolic blood pressure; DBP: diastolic blood pressure; FBS: fasting plasma glucose; TG: triglycerides; TC: total cholesterol; LDL-C : low-density lipoprotein cholesterol; HDL-C : high-density lipoprotein cholesterol; HOMA-IR : homeostasis model assessment-insulin resistance; ^*∗*^*P* < 0.05; ^*∗∗*^*P* < 0.01; ^*∗∗∗*^*P* < 0.001.

## Data Availability

The data will be made available by the corresponding author upon reasonable request.

## Abbreviations

ANGPTL: Angiopoietin-like protein
IR: Insulin resistance
FPG: Fasting plasma glucose
TG: Triglyceride
TC: Total cholesterol
LDL-C: Low-density lipoprotein cholesterol
VLDL: Very-low-density lipoprotein
HDL-C: High-density lipoprotein cholesterol
SBP: Systolic blood pressure
DBP: Diastolic blood pressure
BMI: Body mass index
MetS: Metabolic syndrome
LPL: Lipoprotein lipase
WC: Waist circumference
HC: Hip circumference
ELISA: Enzyme-linked immunosorbent assay
HOMA-IR: Homeostatic model assessment of insulin resistance.
